# Large kidneys predict poor renal outcome in subjects with diabetes and chronic kidney disease

**DOI:** 10.1186/1471-2369-11-3

**Published:** 2010-03-03

**Authors:** Vincent Rigalleau, Magalie Garcia, Catherine Lasseur, François Laurent, Michel Montaudon, Christelle Raffaitin, Nicole Barthe, Marie-Christine Beauvieux, Benoit Vendrely, Philippe Chauveau, Christian Combe, Henri Gin

**Affiliations:** 1 Nutrition-Diabétologie, Centre Hospitalier de Bordeaux and Université de Bordeaux 2-Victor Segalen, Bordeaux (33000), France; 2 Néphrologie and INSERM U889, Centre Hospitalier de Bordeaux and Université de Bordeaux 2-Victor Segalen, Bordeaux (33000), France; 3 Imagerie médicale, Centre Hospitalier de Bordeaux and Université de Bordeaux 2-Victor Segalen, Bordeaux (33000), France; 4 Médecine Nucléaire, Centre Hospitalier de Bordeaux and Université de Bordeaux 2-Victor Segalen, Bordeaux (33000), France; 5 Biochimie, Centre Hospitalier de Bordeaux and Université de Bordeaux 2-Victor Segalen, Bordeaux (33000), France

## Abstract

**Background:**

Renal hypertrophy occurs early in diabetic nephropathy, its later value is unknown. Do large kidneys still predict poor outcome in patients with diabetes and Chronic Kidney Disease (CKD)?

**Methods:**

Seventy-five patients with diabetes and CKD according to a Glomerular Filtration Rate (GFR, by 51Cr-EDTA clearance) below 60 mL/min/1.73 m^2 ^or an Albumin Excretion Rate above 30 mg/24 H, had an ultrasound imaging of the kidneys and were cooperatively followed during five years by the Diabetology and Nephrology departments of the Centre Hospitalier Universitaire de Bordeaux.

**Results:**

The patients were mainly men (44/75), aged 62 ± 13 yrs, with long-standing diabetes (duration:17 ± 9 yrs, 55/75 type 2), and CKD: initial GFR: 56.5 (8.5-209) mL/min/1.73 m^2^, AER: 196 (20-2358) mg/24 H. Their mean kidney lenght (108 ± 13 mm, 67-147) was correlated to the GFR (r = 0.23, p < 0.05). During the follow-up, 9/11 of the patients who had to start dialysis came from the half with the largest kidneys (LogRank: p < 0.05), despite a 40% higher initial isotopic GFR. Serum creatinine were initially lower (Small kidneys: 125 (79-320) μmol/L, Large: 103 (50-371), p < 0.05), but significantly increased in the "large kidneys" group at the end of the follow-up (Small kidneys: 129 (69-283) μmol/L, Large: 140 (50-952), p < 0.005 vs initial). The difference persisted in the patients with severe renal failure (KDOQI stages 4,5).

**Conclusions:**

Large kidneys still predict progression in advanced CKD complicating diabetes. In these patients, ultrasound imaging not only excludes obstructive renal disease, but also provides information on the progression of the renal disease.

## Background

One third of patients with diabetes have kidney damage [1]. The screening of Chronic Kidney Disease (CKD) in patients with diabetes is based on the Albumin excretion rate (AER, threshold: 30 mg/24 H) and the estimated Glomerular Filtration Rate (e-GFR, threshold: 60 mL/min/1.73 m^2^) [2]. Other phenomena occur earlier in the course of diabetic nephropathy, such as glomerular hyperfiltration [3,4], renal hypertrophy [5], and renal histologic lesions [6], but their assessment in day-to-day clinical practice is inconvenient: renal biopsies are invasive, GFR determinations are expensive and GFR estimations are not useful for diagnosis of hyperfiltration [7,8].

Ultrasound allows non-invasive renal imaging at moderate expense, and it is recommended for the first line evaluation of CKD [9], although the discovery of obstructive renal disease or tumors is rare [10]. The overal dimensions of the kidneys can also be determined by ultrasonic imaging [11]. Zerbini et al have recently shown that renal hypertrophy predicts microalbuminuria in patients with type 1 diabetes and normal renal function [12]. Whether kidney size still predicts the progression in more advanced cases, when ultrasound imaging is indicated in clinical practice, is unknown.

In 75 patients with diabetes and CKD, we measured the AER, the GFR by 51Cr-EDTA clearance, and kidney length by ultrasound. The patients were then enrolled in a structured collaborative care program involving diabetologists and nephrologists, and followed up for 5 years, to determine whether their outcome (number of dialysis onset, serum creatinine and e-GFR) differed according to initial kidney size.

## Methods

### Subjects

Seventy-five patients (44 men, mean age 62 ± 13 yrs) were recruited from the Nutrition-Diabetology and Nephrology departments of the Centre Hospitalier Universitaire de Bordeaux. The inclusion criteria were:

1-Diabetes. Twenty patients had type 1 diabetes, 55 type 2,

2-CKD according to an AER above 30 mg/24 H or a GFR below 60 mL/min/1.73 m2, not requiring renal replacement therapy at inclusion,

3-An ultrasound imaging with measurement of the length of the kidneys (Two other patients were excluded because they only had one kidney).

The patients gave written informed consent to participate to the study, which was approved by the ethical committee of our institution. This study was supported by a clinical research program in the Bordeaux University Hospital.

### Analytical methods

The AER was determined on two 24 H urine collections during a short hospitalization, with an immunonephelometric analyzer (Behring Nephelometer 2) using an appropriate kit (Nantiserum VO human albumin, Dade Behring). Serum creatinine was determined on a multiparameter analyzer (Olympus AU 640: Olympus Optical, Tokyo, Japan) using the Jaffé method with bichromatic measurements according to the manufacturer's specifications, and daily calibration of the analyzer. This procedure did not change in our laboratory during the study. The GFR was estimated from serum creatinine by the Mayo clinic quadratic equation [13]. Clearance of the radionucleide marker was measured after intravenous injection of 51Cr-EDTA (Cis Industries, Gif/Yvette, France). All patients were studied in the morning at 9 am, after a light breakfast. After a single bolus of 100 μCi (3.7 MBq) of 51Cr-EDTA, four venous blood samples were withdrawn at 75, 105, 135 and 165 minutes, and urinary samples were collected at 90, 120, 150 and 180 minutes, as previously described [14]. The final result was the mean of the four clearance values. If for one period the urine flow was too low or if a clearance value was not within ± 20% of the mean of the three others, this value was excluded and the mean was calculated for the other three clearances. Less than 5% of the values were excluded this way. The 51Cr-EDTA radioactivity was measured in a gamma counter (COBRA 2, model 05003, Packard Instruments, Meriden, CT). The ultrasound imaging was performed in the department of medical imaging during the same hospitalization., to measure the length of both kidneys.

### Follow-up, care and outcome

This prospective study began on June 2001, untill December 2008. It was based on a cooperative follow-up between diabetologists and nephrologists with the establishment of a joint medical file for each patient. This cooperative follow-up was complementary and included one visit with the diabetologist every four months, and visits with the nephrologist with intervals proportional to the e-GFR. Mean duration of the follow-up was 61 ± 19 (min:1, max:84) months.

The care program objectives included glycemic control according to the French 1999 recommendations (HbA1c < 8.0%, if possible 6.5% without severe hypoglycemia in type 2, and < 7.0% in type 1), but also control of associated factors such as hypertension (objective: < 130/80 mmHg) and dyslipidemia (objective: LDL-cholesterol < 1.3 g/L). We have previously reported [15] that we could obtain significant reductions of HbA1C (-0.7%, 40% of the patients reached the objective), LDL-Cholesterol (-0.4 g/L, 70% of the patients reached the objective), and blood pressure (-10 mmHg systolic, 54% of the patients reached the objective, -5 mmHg diastolic, 95% of the patients reached the objective - 75% were on ACE inhibitors, Ang2 receptor inhibitors, or both), and the Albumin Excretion Rate decreased by -40%. We prescribed 0.8 g protein/kg/d according to the NKF recommendations, except for patients with clinical signs of undernutrition or who were aged 65 years and over.

The primary outcome was requirement for dialysis. The secondary outcomes were the AER, serum creatinine and e-GFR at the end of the follow-up.

### Statistical analysis

The results are expressed as mean ± SD, and median (range) for the GFR, creatinine and AER which were not normally distributed. The association between kidney length and other variables was tested by regression analysis for continuous variables and by ANOVA and unpaired Student's t tests for dichotomous variables. The subjects were then categorized as having large (mean length > median mean length of the group) vs small kidneys, and these two groups were compared by ANOVA for the continuous variables, and by Chi2 for the categorical variables. Non-parametric tests (Wilcoxon, Mann-Witney) were used for the GFR, creatinine and AER. For the primary outcome (dialysis onset), prognostic curves were obtained using the Kaplan-Meier estimation method and compared by log-rank test, the patients who did not present any event (dialysis onset) were censored at the end of the follow-up. A survival analysis using the Cox proportional hazards model was also performed to distinguish the effects of kidney length and GFR on the risk of requiring dialysis. For the secondary outcome, the baseline and final characteristics of the patients were compared by paired Student t tests. Similar analysis were performed after categorizing the subjects as KDOQI stage 1-2,3 and 4-5 [16]. All the analysis were performed using SPSS software, version 10.0. The significance level was fixed at p < 0.05.

## Results

### Baseline characteristics

Seventy five patients were included. Their mean diabetes duration was 17 ± 9 yrs, Body Mass Index: 28.2 ± 5.3, arterial pressure 145 ± 21/80 ± 12 mmHg, and HbA1C: 8.6 ± 1.5%. Their AER was 196 (20-2358) mg/24 H, serum creatinine 126 (50-371) μmol/L, and isotopic GFR was 56.5 (8.5-209) mL/min/1.73 m^2^. Their MCQ e-GFR (63.8 (11-143) mL/min/1.73 m2) was slightly higher (p < 0.05), and was well correlated with isotopic GFR (r = 0.79, p < 0.001).

**Figure 1:**
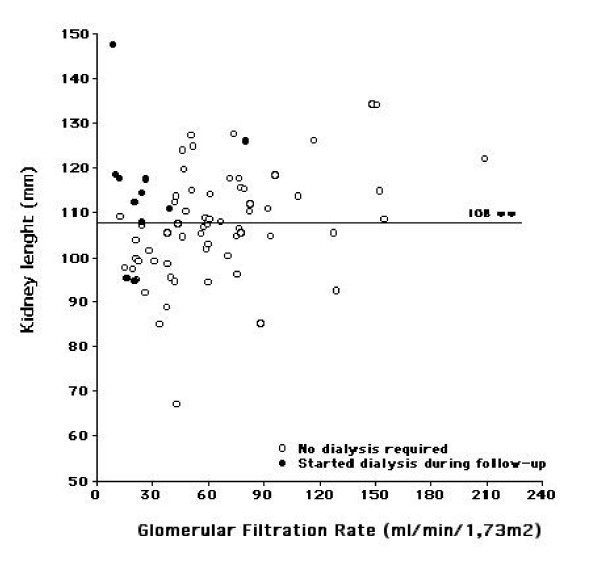
**Mean kidney lenght (mm) as a function (r = 0.23, p < 0.05) of isotopic GFR (mL/min/1.73 m^2^)**. The subjects who required dialysis during the follow-up are depicted as closed circles, the other subjects as open circles.

The lengths of the right (108 ± 13 mm) and left kidney (108 ± 16 mm) did not differ, and were correlated (r = 0.71, p < 0.0001). The analysis was therefore conducted on the mean kidney length (108 ± 13 mm, 67-147), which did not differ for gender or type of diabetes. The mean kidney length was correlated with isotopic GFR (r = 0.23, p < 0.05; figure 1). The correlations of kidney size with age, height, BMI, HbA1C, and Albumin excretion rate were not significant. The kidney lenght were 100 ± 9 mm in normoalbuminuric (n = 6), 106 ± 13 mm in microalbuminuric (n = 39), and 113 ± 11 mm in macroalbuminuric (n = 30) patients (p = 0.056).

### Outcome according to kidney size

**Figure 2:**
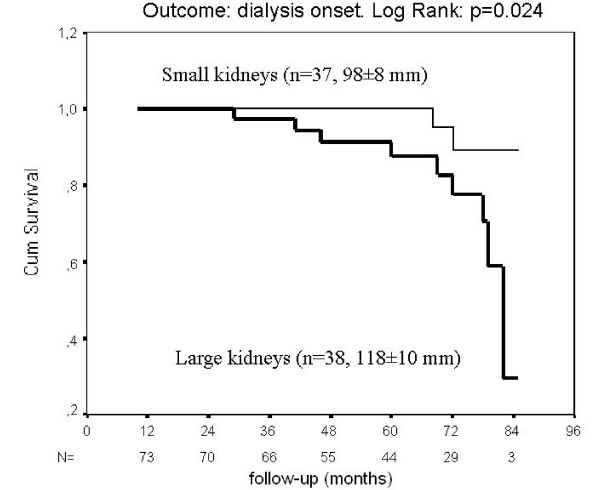
**Survival curves of patients with diabetes and CKD, according to their initial kidney length (end-point: dialysis)**.

The subjects were categorized according to their mean kidney length, as having "large" (> median = 108 mm) or "small" kidneys. The initial characteristics of the two groups did not differ, as shown in table 1, although patients with large kidneys had lower serum creatinine, higher GFR and MCQ e-GFR (all p < 0.05), as would be expected. The higher Albumin Excretion Rate in the "large kidneys" group almost reached significance (p = 0.052). Five years later, 9/11 of the subjects who required dialysis (p = 0.048 by 2-sided Chi2) came from the "large kidneys" group, despite their higher initial GFR; these subjects are depicted as closed circles on the figure 1. The survival curve (end-point: dialysis) is depicted on the figure 2 (LogRank: p = 0.024). The serum creatinine increased in the "large kidneys" group (p = 0.002), whereas it kept stable in the "small kidneys" group.

**Table 1:** Baseline and final characteristics of the subjects according to their initial mean kidney length (threshold = median = 108 mm).

	Small	Large
N	37	38

**Initial**		
Mean kidney length (mm)	98 ± 8	118 ± 10***
Age (yrs)	64 ± 12	61 ± 14
Gender (%men)	54%	63%
BMI	27.6 ± 6.0	28.7 ± 4.5
HbA1C (%)	8.8 ± 1.5	8.4 ± 1.4
Diabetes duration (yrs)	17 ± 10	16 ± 9
Blood pressure (mmHg)	147 ± 19/82 ± 12	143 ± 23/77 ± 13
AER (mg/24 H)	112 (20-2358)	390 (28-2312)
Serum creatinine (μmol/L)	125 (79-320)	103 (50-371)*
MCQ e-GFR (mL/min/1.73 m2)	58 (17-109)	73 (26-143)*

Isotopic GFR(mL/min/1.73 m2)	43(15-129)	71(8.5-209)*

**End of the follow-up**		

Duration of follow-up (months)	61 ± 20	60 ± 19

Serum creatinine (μmol/L)	129 (69-283)	140 (50-952)

Decline in MCQ e-GFR (mL/min/1.73 m2/yr)	-2.1 (-10-+12)	-2.7 (-28-+16)

Subjects on hemodialysis	2	9*

* for p < 0.05.

The eleven subjects who required dialysis during the follow-up had lower isotopic GFR (20.4 (8.5-80) vs 59.2 (12-209) mL/min/1.73 m2 for others, p = 0.001) and larger kidneys (116 ± 19 vs 107 ± 12 mm, p = 0.042) on inclusion. The multivariate survival analysis using the Cox proportional hazards model showed that both initial GFR (ExpB = 0.94, p = 0.005) and mean kidney length (ExpB = 1.05, p = 0.02) were predictors of dialysis onset. The influence of the kidney lenght did not remain significant after adjustment for the AER.

There was no significant difference between the subjects with type 1 and type 2 diabetes (gender, GFR, Albumin Excretion Rate, kidney lenght, HbA1C, known duration of diabetes), except for the age and the BMI as expected. Although the different outcomes only reached significance for dialysis onset for type 2, according to the restricted number of patients, the results seemed homogenous in both types of diabetes, as shown in the table 2

**Table 2:** The renal outcome of the subjects according to their type of diabetes and kidney size.

	Type 1 diabetes		Type 2 diabetes	
n	20		55	

age	54 ± 17		65 ± 10°°°	

BMI	23.6 ± 3.4		29.8 ± 4.9°°°	

	Small-108 mm-Large		Small-108 mm-Large	

n	10	10	27	28

Isotopic GFR (mL/min/1.73 m^2^)	59 (20-94)	73 (12-209)	40 (15-129)	60* (8-154)

Albumin Excretion Rate (mg/24 H)	98 (20-1080)	196 (32-2312)	137 (20-2358)	390 (28-2010)

Decline of e-GFR (mL/min/1.73 m^2^/yr)	-0.5 (-6-+8)	-2.0 (-8-+16)	-1.1 (-1-+12)	-2.0 (-28-+3)

Dialysis onset	1	2	1	7*

* indicates p < 0.05 vs small kidneys. °°° indicates p < 0.001 vs Type 1.

### Kidney size and outcome according to the stratification of CKD

As shown in table 3, the kidneys tended to be smaller in the most advanced stages of CKD (p = 0.08). The differences in progression reached significance only in the severe renal failure groups, where all the six subjects who had kidneys > 112 mm had to start dialysis. The decline of e-GFR did not significantly correlate to the kidney size (r = 0.15, p = 0,20). Twenty-one subjects had microalbuminuria (30-300 mg/24 H) and an isotopic GFR > 60 mL/min/1.73 m^2^; none of them required dialysis during the follow-up. Although the difference was not significant, the MCQ-eGFR declined faster for the eleven subjects with the largest (> 109 mm) kidneys: -4.2 mL/min/1.73 m^2 ^(-14-+16), vs -2.3 (-6-+5) for the 10 subjects with the smaller kidneys.

**Table 3:** The renal outcome of the subjects according to their initial stratification of CKD (Severe renal failure = GFR < 30, Moderate renal failure = GFR 30-60, Kidney damage = GFR > 60 mL/min/1.73 m^2 ^and AER > 30 mg/24 H), and their mean kidney length.

	Severe renal failure (CKD4)		Moderate renal failure (CKD3)		Kidney damage (CKD 1-2)	
n	19		25		31	

AER (mg/24 H)	526 (23-2010)		137 (20-2312)		148 (32-2358)	

Isotopic GFR (mL/min/1.73 m^2^)	20.7 (8.5-28)		46.1 (30-59)		80.2 (60-209)	

Median kidney length (mm)	104 (92-147)		107 (67-127)		112 (85-134)	

	Small-104 mm-Large		Small-107 mm-Large		Small-112 mm-Large	

n	10	9	13	12	16	15

Decline of e-GFR (mL/min/1.73 m^2^/yr)	+0.3 (-9-+8)	-2.0* (-14--1)	-2.1 (-10-+12)	-2.0 (-9-+3)	-3.0 (-8-+5)	-2.8 (-28-+16)

Dialysis onset	2	7*	0	1	0	1

* indicates p < 0.05 vs small kidneys.

## Discussion

Renal hypertrophy with ~30% higher than normal kidney size has been noted in type 1 diabetics [17], in some cases after only a few days of hyperglycemia [18]. Although less common [19] and less pronounced [20], it also exists in type 2 diabetes [21]. Since the pioneer work of Mogensen [22,23], the high risk of microalbuminuria in diabetic patients with large kidneys has been reported frequently [24-26]. This was recently emphazised by Zerbini et al, who demonstrated a threefold incidence of microalbuminuria and almost threefold faster decline in GFR during the 9 years follow-up of the highest quartile of ultrasound-measured kidneys of normoalbuminuric type 1 diabetic subjects [12]. However, all these studies were on the initial stages of renal involvement. In humans, diabetic renal hypertrophy can persist for years despite good glucose control [18,27-29]. But with advanced renal insufficiency the kidneys become smaller [30], and this also occurs in diabetic patients, as reflected by the correlation between GFR and renal size as we found, in line with other authors [12,28]. This late course raised the issue of whether kidney size remained a marker of progression in diabetic patients with more advanced CKD.

Our inclusion criterion was CKD: most of our patients had an isotopically determined GFR below 60 mL/min/1.73 m2, and the others had an AER above 30 mg/24 H. The half with the largest kidneys did not differ from the others in age, gender, diabetes duration, BMI, and the conventional progression factors (blood pressure, AER, HbA1C) were not significantly higher. They had 40% higher GFR than the "small kidney" group, as expected. High GFR has been associated with faster rates of decline in renal function in other cohorts [31,32]. The risk of requirement for dialysis was also associated with kidney size after accounting for initial GFR. Clearly the higher GFR in the "large kidney" group could not contribute to the higher rate of dialysis onset, and the worst outcome with large kidneys was still apparent after stratifying the patients for CKD. Baumgartl et al also found higher serum creatinine and more frequent dialysis onset in 37 patients with "large kidneys" during the long term follow-up of 73 type 1 diabetic patients with initially normal creatinine [26]. This is in line with our findings, although an important difference is that we investigated patients with CKD: the prognostic value of kidney size for these more advanced patients has not been reported to date. We feel that this result is of interest, as patients with CKD are likely to undergo an ultrasound imaging in day-to-day clinical practice. Once the possibility of obstructive renal disease has been excluded, about 99% of the ultrasound imaging reports are classified as normal, whereas they contain interesting information about the later outcome of the patients.

Renal hypertrophy is an early phenomenon in diabetic patients: clinical observations show that it precedes microalbuminuria [12], which is now recognized as a non-obligatory step as ~20% of renal insufficient diabetic patients are normoalbuminuric [33-35]. Baumgartl indeed mentioned a few cases of renal hypertrophy preceeding severe renal disease without any abnormal AER [26]. The underlying pathological abnormalities are enlarged glomerular volume, which may not be similar in type 1 and type 2 diabetes [36], and tubular enlargement [37]; experimental studies suggest that the renal hypertrophy may be due to the inhibition of kidney AMP-activated protein kinase by high glucose level [38], and that it precedes glomerular hyperfiltration [39], giving rise to it by a tubuloglomerular feedback mechanism [40]. Although the reversal of renal hypertrophy has been observed in animals with various treatments, such as glucose control [41], neutralizing VEGF antibody [42] or C-peptide [43], human observations are in favor of a persistence of renal hypertrophy [17,27-29], but its outcome has yet to be reported in advanced stages of diabetic renal disease.

Two limitations of our study should be born in mind. Apart from the "hard" endpoint of dialysis onset, we used an estimation of GFR to measure the decline in renal function; creatinine-based estimations (Cockcroft-Gault, MDRD) are known to underestimate GFR decline in diabetes [44], especially in patients with mild to moderate renal impairment [32]. For this reason we used the Mayo Clinic Quadratic equation that is less biased according to GFR [45] and less affected by the progression-related biases [46]; the rates of e-GFR declines however differed significantly only in the "severe CKD" group (for the comparison between "small" and "large" kidneys on the whole group, p = 0.10). Measuring GFR would presumably have led to greater differences in the less advanced stages, as reported in the 68 patients who were evaluated twice by Zerbini et al [12]. Another limitation was that kidney length was measured ultrasonographically by several observers. Nevertheless, this has been found to be a reliable method with an acceptable magnitude of variation when made by a single or different ultrasonographers [47]. The kidney lenght is correlated to the renal volume: it is indeed included in the ellipsoid formula to calculate it [48]. Neither the side (right or left) nor the patient position (prone or supine) had a significant influence on this measurement, and its good reproducibility has also been reported in patients with large kidneys due to polycystic kidney disease [49]. Further work will be necessary to confirm our findings in other cohorts, and to determine whether the threshold of kidney length that we found is a reproducible indicator of progression according to the level of renal function.

## Conclusions

Large kidneys still predict progression in advanced CKD complicating diabetes. In these patients, ultrasound imaging not only excludes obstructive renal disease, but also provides information on the progression of the renal disease,

## Competing interests

The authors declare that they have no competing interests.

## Authors' contributions

VR conceived of the study, and participated in its design and coordination and wrote the manuscript. MG collected data and performed the statistical analysis. CL, CR, BV, PC collected data and participated in the study design. FL and MM performed the ultrasound imaging, NB performed the isotopic GFR measurements, MCB performed the biochemical analysis. CC and HG conceived of the study, and participated in its design and coordination. All authors read and approved the final manuscript.

## Pre-publication history

The pre-publication history for this paper can be accessed here:

http://www.biomedcentral.com/1471-2369/11/3/prepub
